# Global burden of fall-related injuries attributable to low bone mineral density in women aged 50–69 years: inequalities, projections to 2050, and Mendelian randomisation

**DOI:** 10.7189/jogh.16.04180

**Published:** 2026-06-19

**Authors:** Bo Zhang, Qiaojie Chen, Yang Chen, Haijun Zhang

**Affiliations:** Department of Orthopaedics, Ningbo No. 2 Hospital, Wenzhou Medical University, Ningbo, China

## Abstract

**Background:**

Low bone mineral density (LBMD) increases fragility fracture risk and may contribute importantly to disability after falls. Within the Global Burden of Disease (GBD) framework, the attributable fall-related burden is better interpreted as fracture susceptibility and post-fall disability than as a direct effect on the occurrence of falls. However, the magnitude, inequality, and future trajectory of this burden in women aged 50–69 years remain unclear.

**Methods:**

Using GBD 2021 estimates from 1990–2021, we quantified LBMD-attributable fall-related disability-adjusted life years (DALYs), years lived with disability (YLDs), and age-standardised rates in women aged 50–69 years. Men were included for contextual comparison. We assessed temporal trends, socioeconomic inequality, frontier gaps, burden decomposition, and conditional projections to 2050. We performed two-sample Mendelian randomisation to examine associations of genetically predicted femoral-neck bone mineral density with falls and femoral fractures.

**Results:**

From 1990–2021, age-standardised DALY and YLD rates declined modestly, whereas absolute DALYs nearly doubled and YLDs increased markedly. In 2021, women had approximately 35–45% higher age-standardised disability-adjusted life-year rate (ASDR) and about 75% more YLDs than men aged 50–69 years. Relative inequality, as measured by the concentration index, was directionally positive but statistically uncertain, whereas absolute inequality remained substantial. Conditional projections suggested further declines in age-standardised rates by 2050 but continued growth in absolute disability burden. Genetically predicted lower femoral-neck bone mineral density showed no clear association with falls (inverse-variance weighted odds ratio (IVW OR) = 1.29; 95% CI = 0.86, 1.95) but was associated with higher risk of fracture of femur (OR = 1.71; 95% CI = 1.37, 2.14).

**Conclusions:**

LBMD-attributable fall-related injury burden in women aged 50–69 years remains a substantial and unequally distributed source of disability. The GBD and Mendelian randomisation findings suggest that LBMD contributes more strongly to fracture susceptibility and disability after falls than to fall occurrence itself, supporting earlier osteoporosis risk assessment and integrated bone-, muscle-, and fall-prevention strategies.

Low bone mineral density (LBMD) is an established risk factor for fragility fractures and an important determinant of injury severity after falls [1,2]. Postmenopausal women are particularly vulnerable because oestrogen loss during the menopausal transition accelerates bone loss and increases fracture risk from the mid-50s onward [3,4]. In this population, falls commonly precipitate osteoporotic fractures, leading to substantial morbidity, mortality, and economic costs [5,6].

Falls that culminate in fracture are increasingly understood as failures of an integrated muscle-bone unit rather than isolated skeletal events [7,8]. Around the menopausal transition, declining femoral-neck bone mineral density (BMD) occurs alongside worsening muscle strength and performance, creating a midlife ‘osteosarcopenia window’ in which falls are more likely to result in fracture, prolonged recovery, and disability [3,7,9–11]. LBMD captures the skeletal component of this process, but fall-related disability is jointly shaped by skeletal fragility, neuromuscular function, and environmental context [1,7,10,11].

The global burden of fractures reached 25.8 million YLDs in 2019, a 65% increase since 1990, with older women disproportionately affected [8]. The period from 50–69 years is a critical window for intervention because many women first enter sustained bone loss, declining physical performance, and rising fracture susceptibility during these years [1,10]. Prevention initiated at this stage may avert substantial later-life disability.

Despite this clinical importance, most global epidemiological assessments have focused on adults older than 60 or 70 years or on both sexes combined [8,12,13]. Limited work has specifically examined women aged 50–69 years, when preventive efforts may still alter long-term trajectories [3,14]. Recent Global Burden of Disease (GBD) 2021 analyses described the overall burden and projections of falls among midlife women [15] and quantified attributable burden for major risk factors, including LBMD, across populations [16]. However, they did not specifically characterise LBMD-attributable fall-related injury burden in women aged 50–69 years while jointly evaluating inequality, frontier gaps, and the distinction between fall occurrence and post-fall fracture-related disability.

We therefore characterised global, regional, and national patterns in LBMD-attributable fall-related injury burden among women aged 50–69 years from 1990 to 2021. We assessed temporal trends, sociodemographic inequalities, frontier gaps, burden decomposition, and conditional projections to 2050. To complement these burden estimates, we also conducted two-sample Mendelian randomisation (MR) analyses of femoral-neck BMD on FinnGen falls and femoral fractures, treating MR as complementary triangulation rather than one-to-one validation of the GBD analyses.

**Adherence to JoGH’s Guidelines for Reporting Analyses of Big Data Repositories Open to the Public (GRABDROP)**

This secondary analysis of publicly available Global Burden of Disease and FinnGen data was conducted and reported in accordance with the Strengthening the Reporting of Observational Studies in Epidemiology (STROBE) statement and JoGH’s Guidelines for Reporting Analyses of Big Data Repositories Open to the Public (GRABDROP) [17,18]. The completed GRABDROP table and STROBE checklist are provided in Table S5 and S6 in the **Online Supplementary Document**.

## METHODS

### Study design and data source

In this retrospective observational study, we used publicly available data from the Global Burden of Disease (GBD) Study 2021, which covers 204 countries and territories [19]. We extracted estimates for women aged 50–69 years from 1990–2021, representing the perimenopausal and early postmenopausal period when bone loss accelerates. Men aged 50–69 years were included for contextual comparison only. Analyses were conducted at global, regional, national, and socio-demographic index (SDI) quintile levels. Because the study used only publicly available, de-identified and aggregated data, ethics committee approval was not required.

### Exposure and outcome definitions

The exposure was LBMD, modelled as a continuous risk factor within the GBD 2021 comparative risk assessment. LBMD was defined as the age- and sex-specific shortfall of femoral-neck dual-energy x-ray absorptiometry (DXA)-measured bone mineral density (BMD) from the theoretical minimum-risk exposure level, set at the 99th percentile of the National Health and Nutrition Examination Survey reference distribution [20]. This standardised reference supports cross-country comparability but is not a universally representative global normative distribution; alternative reference distributions would mainly affect the absolute magnitude of attributable estimates. Attributable burden was estimated using population attributable fractions (PAFs), which in the GBD framework represent modelled comparative-risk quantities rather than direct individual-level causality [16,21]. For multifactorial outcomes such as falls, these estimates should therefore be interpreted cautiously as partly reflecting fracture susceptibility and post-fall disability rather than fall occurrence itself. The outcome was unintentional falls, coded in the International Classification of Diseases, 10th Revision (ICD-10) as W00-W19 [19].

### Outcome measures

The primary measures were age-standardised DALY rates (ASDR) and age-standardised YLD rates (ASYR) per 100 000 population, representing total health loss and non-fatal burden from LBMD-attributable fall-related injuries within the GBD framework. Rates were standardised to the GBD reference population, and absolute counts are presented with 95% uncertainty intervals (UIs) derived from 1000 posterior draws.

### Statistical analysis

#### Temporal trends

We estimated annual changes in age-standardised rates using weighted least-squares regression of the natural logarithm of the rate on calendar year and calculated estimated annual percentage changes (EAPCs) with 95% confidence intervals (CIs) [22]. Restricted cubic splines were used to assess nonlinearity, and model fit was compared using the Akaike Information Criterion [23].

#### Socioeconomic inequality

Countries and territories were ranked by SDI from lowest to highest. Absolute inequality was assessed using the slope index of inequality (SII), and relative inequality using the concentration index (CIX) [24,25]. Concentration curves were generated by plotting cumulative population share against cumulative outcome share, and 95% confidence bands were estimated by bootstrap resampling with 1000 iterations [26].

### Frontier analysis

Frontier analysis benchmarked country performance against the lowest observed burden at a given SDI. The empirical frontier was estimated using locally estimated scatterplot smoothing (LOESS)-based lower-envelope smoothing, with constrained cubic splines used to preserve a non-increasing SDI-frontier relation [26,27]. Frontier gaps were defined as the vertical differences between observed and frontier rates at the same SDI, with uncertainty estimated by bootstrap resampling. These gaps were interpreted as descriptive inefficiency gaps rather than causal effects.

### Burden decomposition

We applied Das Gupta decomposition to partition changes in absolute burden into contributions from population growth, population ageing, and changes in age-specific rates [28]. Uncertainty in the decomposed contributions was estimated by Monte Carlo simulation with 10 000 iterations [29].

### Bayesian projections

To project outcomes to 2050, we used Bayesian age-period-cohort models estimated by Integrated Nested Laplace Approximations [30]. Forecasts were conditioned on United Nations World Population Prospects 2022 medium-variant demographics [31], and uncertainty was summarised using 50%, 80%, and 95% CIs. To assess robustness, we repeated projections using 2035, 2040, and 2050 forecast horizons. These estimates were interpreted as conditional projections rather than deterministic forecasts because future treatment uptake, screening access, obesity trends, and health-system changes may alter trajectories [32–34].

### Mendelian randomisation

We performed two-sample Mendelian randomisation (MR) to assess associations of genetically predicted femoral-neck BMD with falls and fracture of femur. Genetic instruments were obtained from the Medical Research Council Integrative Epidemiology Unit (MRC IEU) Open Genome-Wide Association Studies (OpenGWAS) database (ieu-a-980) and clumped at r^2^<0.001 within 10 Mb, yielding 21 independent single-nucleotide polymorphisms [35]. Outcome summary statistics were from FinnGen Release 10 (FinnGen consortium, Institute for Molecular Medicine Finland, University of Helsinki, Helsinki, Finland) for medically recorded falls and fracture of femur [36–38]. Because these outcomes were not restricted to women aged 50–69 years and are not identical to GBD-attributed disability burden, MR was interpreted as complementary triangulation rather than direct one-to-one validation. Inverse-variance weighted (IVW) analysis was primary; MR-Egger, weighted median, and weighted mode were used as sensitivity analyses. We also assessed heterogeneity, directional pleiotropy, leave-one-out influence, and instrument strength.

### Software and statistical considerations

Analyses were performed in *R*, version 4.4.2 (R Core Team, Vienna, Austria) using ‘mgcv,’ ‘boot,’ ‘INLA,’ and ‘TwoSampleMR’ (version 0.6.29), together with ‘JD_GBDR v2.37.’ The executable bundle and scripts for the frontier analysis, decomposition, and Bayesian age-period-cohort (BAPC) projections are available from the corresponding author upon reasonable request. Two-sided *P* < 0.05 was considered statistically significant.

## RESULTS

### Global temporal trends (1990–2021)

The global absolute LBMD-attributable fall-related injury burden in women aged 50–69 years increased markedly from 1990 to 2021 (Table S1 in the **Online Supplementary Document**). DALYs rose 93.0%, from 1 083 598 (95% UI = 853 310, 1 343 612) in 1990 to 2 091 475 (95% UI = 1 639 042, 2 629 110) in 2021. YLDs increased 91.8%, from 737 417 (95% UI = 519 562, 989 220) to 1 414 595 (95% UI = 989 690, 1 907 214).

Age-standardised rates declined only modestly (**Table 1**). ASDR fell from 313.57 (95% UI = 246.93, 388.81) to 285.54 (95% UI = 223.77, 358.94) per 100 000, and ASYR from 213.39 (95% UI = 150.35, 286.26) to 193.13 (95% UI = 135.12, 260.38) per 100 000. The corresponding EAPCs were –0.50% per year for ASDR and –0.52% per year for ASYR, indicating gradual rate improvement despite population growth and ageing.

**Table 1:** Age-standardised rates (ASDR, ASYR) of LBMD-attributable fall-related injury burden among women aged 50–69 y, by global total, five SDI quintiles, and 21 GBD regions

		ASDR			ASYR	
**Location**	1990, per 100 000 (95% UI)	2021, per 100 000 (95% UI)	EAPC (95% CI)	1990, per 100 000 (95% UI)	2021, per 100 000 (95% UI)	EAPC (95% CI)
Global	313.57 (246.93, 388.81)	285.54 (223.77, 358.94)	−0.50(−0.59, −0.41)	213.39 (150.35, 286.26)	193.13 (135.12, 260.38)	−0.52(–0.61, –0.43)
High SDI	333.36 (241.82, 440.42)	346.63 (251.64, 462.70)	0.18 (0.09, 0.27)	285.39 (197.28, 392.85)	299.26 (205.95, 415.52)	0.18 (0.11, 0.26)
High-middle SDI	291.43 (219.02, 375.50)	239.22 (177.89, 318.20)	−1.06(−1.22, −0.90)	238.79 (167.99, 323.52)	198.45 (136.46, 274.44)	−1.02(−1.18, −0.86)
Middle SDI	259.92 (209.25, 313.09)	240.94 (187.24, 299.82)	−0.63(−0.85, −0.42)	156.14 (112.25, 205.63)	153.99 (109.32, 207.18)	–0.44(–0.71, –0.18)
Low-middle SDI	407.99 (320.33, 494.23)	360.26 (289.04, 431.70)	−0.49(−0.57, −0.40)	189.66 (135.57, 249.02)	171.00 (123.71, 225.91)	−0.48(−0.57, −0.39)
Low SDI	323.12 (258.37, 389.78)	298.37 (241.60, 360.42)	−0.26(−0.33, −0.19)	121.00 (87.20, 159.42)	125.69 (90.89, 165.15)	0.12 (0.05, 0.19)
Andean Latin America	132.33 (103.78, 161.87)	140.60 (108.59, 177.04)	0.10 (0.05, 0.16)	86.80 (62.41, 114.90)	98.17 (68.84, 133.21)	0.33 (0.22, 0.43)
Australasia	340.64 (237.29, 466.86)	352.05 (244.69, 480.60)	0.29 (0.20, 0.39)	312.96 (209.83, 440.20)	321.70 (215.57, 448.51)	0.22 (0.12, 0.32)
Caribbean	126.63 (99.91, 155.31)	136.15 (105.85, 170.78)	0.12 (0.03, 0.20)	80.71 (57.74, 109.20)	93.08 (65.90, 125.59)	0.43 (0.26, 0.59)
Central Asia	180.96 (135.41, 232.14)	140.17 (103.15, 184.93)	−0.96 (−1.02, −0.89)	145.45 (101.40, 196.24)	117.74 (81.38, 161.27)	−0.84 (−0.91, −0.77)
Central Europe	398.24 (305.03, 506.91)	290.21 (210.70, 387.45)	−1.23 (−1.33, −1.13)	311.72 (220.70, 418.95)	247.94 (170.39, 345.57)	−0.91 (−0.97, −0.84)
Central Latin America	275.32 (214.51, 345.96)	168.51 (126.48, 215.61)	−1.17 (−1.40, −0.94)	210.98 (151.76, 281.29)	138.40 (96.69, 185.33)	−0.81 (−1.10, −0.52)
Central sub-Saharan Africa	199.03 (154.01, 251.10)	189.58 (145.79, 244.75)	−0.27 (−0.31, −0.23)	60.04 (43.60, 78.84)	64.33 (46.84, 85.06)	0.11 (0.06, 0.16)
East Asia	222.64 (171.43, 283.67)	204.29 (153.08, 268.58)	−0.79 (−1.25, −0.33)	160.27 (115.02, 213.90)	161.23 (111.73, 222.83)	−0.67 (−1.35, 0.00)
Eastern Europe	327.92 (240.64, 432.92)	305.11 (223.35, 409.44)	−0.85 (−1.26, −0.45)	284.70 (198.30, 388.82)	257.79 (176.51, 362.57)	−0.81 (−1.17, −0.46)
Eastern sub-Saharan Africa	222.06 (171.74, 278.43)	179.97 (146.41, 217.06)	−0.84 (−0.90, −0.78)	65.08 (47.50, 86.09)	62.40 (45.27, 82.59)	−0.18 (−0.22, −0.14)
High-income Asia Pacific	260.12 (184.31, 350.53)	229.54 (159.96, 314.40)	−0.48 (−0.64, −0.31)	233.50 (159.10, 323.60)	213.05 (145.02, 297.80)	−0.35 (−0.52, −0.18)
High-income North America	284.34 (203.86, 379.62)	406.77 (299.36, 550.84)	1.37 (1.04, 1.71)	250.67 (172.90, 346.92)	341.25 (235.64, 484.43)	1.16 (0.79, 1.54)
North Africa and Middle East	147.11 (111.16, 189.15)	135.47 (100.15, 176.97)	−0.48 (−0.58, −0.37)	106.38 (75.17, 143.01)	109.22 (75.65, 151.38)	−0.12 (−0.24, 0.01)
Oceania	213.52 (161.05, 277.90)	254.29 (191.02, 334.91)	0.49 (0.45, 0.52)	162.71 (117.16, 217.75)	208.61 (151.22, 279.35)	0.68 (0.63, 0.73)
South Asia	561.74 (436.17, 679.87)	513.31 (406.51, 611.67)	−0.43 (−0.53, −0.33)	242.40 (173.70, 318.30)	230.73 (167.82, 305.24)	−0.32 (−0.40, −0.25)
Southeast Asia	243.56 (189.00, 293.71)	193.59 (154.45, 236.00)	−1.22 (−1.41, −1.04)	114.19 (82.06, 151.40)	104.52 (75.30, 139.58)	−0.69 (−0.84, −0.55)
Southern Latin America	221.77 (163.97, 285.82)	223.22 (158.99, 298.69)	0.07 (−0.04, 0.19)	190.34 (133.89, 253.89)	202.49 (138.33, 277.56)	0.20 (0.06, 0.35)
Southern sub-Saharan Africa	76.99 (59.46, 98.52)	61.23 (46.94, 78.63)	−0.76 (−0.82, −0.69)	54.41 (38.92, 73.66)	40.22 (28.55, 55.00)	−1.07 (−1.14, −1.01)
Tropical Latin America	263.84 (198.05, 335.00)	245.11 (188.29, 308.94)	−0.41 (−0.54, −0.28)	218.89 (155.17, 289.28)	188.47 (133.54, 251.97)	−0.70 (−0.92, −0.47)
Western Europe	397.63 (287.71, 529.50)	384.81 (273.15, 516.97)	0.03 (−0.05, 0.11)	341.03 (234.52, 471.53)	342.00 (232.55, 473.44)	0.11 (0.01, 0.22)
Western sub-Saharan Africa	190.33 (152.77, 225.58)	163.32 (130.87, 199.61)	−0.53 (−0.57, −0.50)	73.02 (53.19, 96.03)	69.47 (50.44, 91.60)	−0.14 (−0.21, −0.07)

ASDR – age-standardised DALY rate, ASYR – age-standardised YLD rate, CI – Confidence Interval, EAPC – estimated annual percentage change, GBD – Global Burden of Disease, LBMD – Low bone mineral density, SDI – socio-demographic index, UI – uncertainty interval

In the GBD comparative risk assessment for falls among women aged 50–69 years in 2021, LBMD was the largest risk factor included (Figure S1 in the **Online Supplementary Document**). This ranking should be interpreted within the GBD attribution framework rather than as evidence that LBMD directly drives the occurrence of falls.

### Sex- and age-specific patterns

For contextual comparison, men aged 50–69 years had a lower burden than women throughout the study period (Figure S2, Panel A and B in the **Online Supplementary Document**). In 2021, women accounted for 2.09 million DALYs and 1.41 million YLDs, compared with 1.44 million DALYs and 0.81 million YLDs in men. Women also had approximately 35–45% higher ASDR and about 75% more YLDs.

Burden increased steeply with age in women (Figure S2, Panel C and D in the **Online Supplementary Document**). Global DALY rates rose from 129.3 per 100 000 (95% UI = 97.3, 171.8) in those aged 50–54 years to 565.4 per 100 000 (95% UI = 445.2, 704.4) in those aged 65–69 years. South Asia had the highest DALY rate in women aged 65–69 years, whereas Southern sub-Saharan Africa had the lowest rates in women aged 50–54 years.

### Geographic variations and national patterns

**Figure 1:**
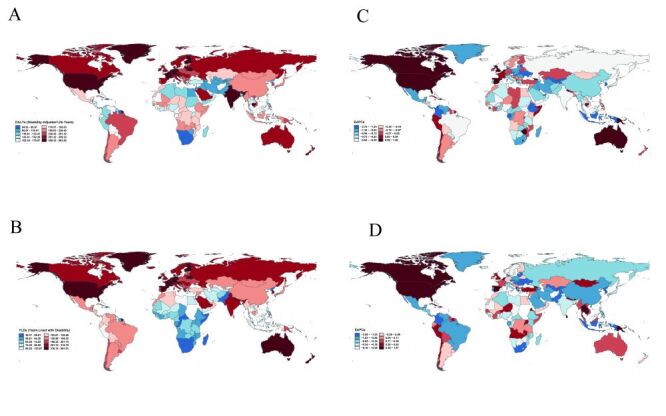
Global distribution and temporal change of LBMD-attributable fall-related injury burden in women aged 50–69 years. **Panel A.** Age-standardised DALY rate (ASDR) per 100 000 population in 2021. **Panel B.** Age-standardised YLD rate (ASYR) per 100 000 population in 2021. **Panel C.** Estimated annual percentage change (EAPC) in ASDR, 1990–2021. **Panel D.** EAPC in ASYR, 1990–2021. ASDR – age-standardised disability-adjusted life year rate, ASYR – age-standardised years lived with disability rate, DALY – disability-adjusted life year, EAPC – estimated annual percentage change, LBMD – low bone mineral density, YLD – years lived with disability.

Geographic disparities were substantial in 2021 (**Figure 1**, Panel A and B). ASDR were highest in Belgium, Andorra, and India, and lowest in South Africa, the USA Virgin Islands, and Saint Vincent and the Grenadines. ASYR patterns largely mirrored those of DALYs, with the highest rates in Andorra, Belgium, and Finland, and the lowest rates in South Africa, Eswatini, and Lesotho.

National trends from 1990 to 2021 were heterogeneous (**Figure 1**, Panel C and D). The fastest increases in ASDR occurred in the USA, the Netherlands, and Puerto Rico, whereas the steepest declines occurred in Latvia, Hungary, and Armenia.

### Sociodemographic gradient

Higher observed burden was associated with higher sociodemographic development (Figure S3, Panels C and D in the **Online Supplementary Document**). At the country level in 2021, both outcomes were positively associated with SDI, with a moderate correlation for ASDR (Spearman’s r = 0.31; 95% CI = 0.16, 0.46, *P* < 0.001) and a stronger correlation for ASYR (r = 0.67; 95% CI = 0.57, 0.76, *P* < 0.001). Country scatterplots showed a stepwise rise across SDI quintiles.

Regional patterns across the 21 GBD regions were broadly similar (Figure S3, Panel A and B in **the** **Online Supplementary Document**), again with a stronger SDI gradient for ASYR than for ASDR. Over time, trajectories differed by region: ASDR rose in high-income North America but declined in South Asia, while East Asia showed a U-shaped pattern in ASYR. Overall, these findings indicate persistent cross-regional differences with limited convergence in some lower-SDI settings.

### Inequality metrics

**Figure 2:**
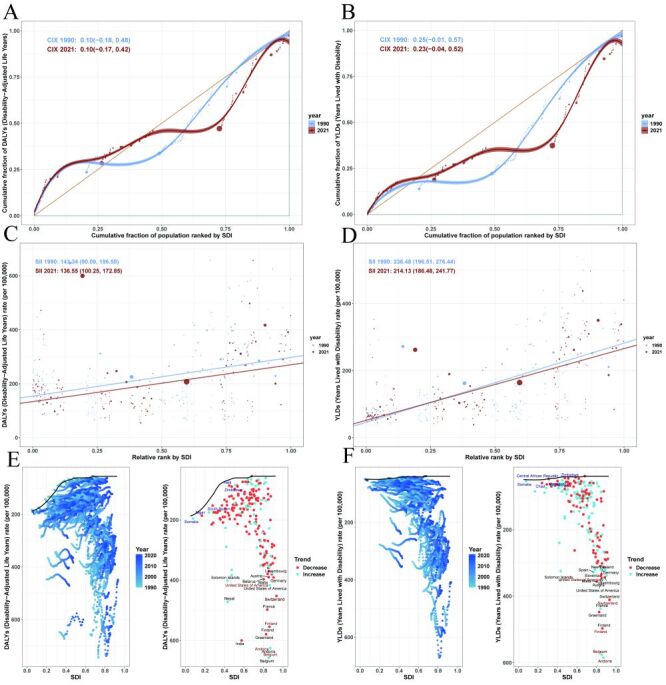
Socio-demographic inequality and frontier benchmarking of LBMD-attributable fall-related injury burden in women aged 50–69 years, 1990–2021. **Panel A.** Concentration curves for age-standardised DALY rates by socio-demographic index (SDI) in 1990 and 2021; the 45° line indicates equality, and the area between each curve and the equality line yields the concentration index (CIX). **Panel B.** Concentration curves for age-standardised YLD rates by SDI in 1990 and 2021. **Panel C.** Slope index of inequality (SII) for age-standardised DALY rates in 1990 and 2021; points represent countries and fitted lines represent population-weighted linear regressions of rates on relative SDI rank (0 = lowest SDI, 1 = highest SDI). **Panel D.** SII for age-standardised YLD rates in 1990 and 2021. **Panel E.** Frontier benchmarking of age-standardised DALY rates against SDI, 1990–2021; left, country trajectories coloured by year; right, 2021 country estimates coloured according to whether rates increased or decreased from 1990 to 2021. **Panel F.** Frontier benchmarking of age-standardised YLD rates against SDI, 1990–2021; left, country trajectories coloured by year; right, 2021 country estimates coloured according to whether rates increased or decreased from 1990 to 2021. In Panels E and F, the black curve indicates the empirical frontier (lowest observed burden at a given SDI), and the vertical distance to the frontier represents the frontier gap. Selected countries are labelled. ASDR – age-standardised disability-adjusted life year rate, ASYR – age-standardised years lived with disability rate, CI – confidence interval, CIX – concentration index, DALY – disability-adjusted life year, SDI – socio-demographic index, SII – slope index of inequality, YLD – years lived with disability.

Quantitative inequality metrics suggested that a greater share of the observed burden was borne by higher-SDI countries, but relative inequality remained statistically uncertain (**Figure 2**, Panel A and B). Concentration indices were positive from 1990 to 2021. DALYs showed CIX = 0.10 (95% CI = −0.18, 0.48) in 1990 and 0.10 (95% CI = −0.17, 0.42) in 2021, while YLDs showed CIX = 0.25 (95% CI = −0.01, 0.57) and 0.23 (95% CI = −0.04, 0.52), respectively. Because these intervals included zero, the CIX results are better read as evidence of a directional tendency toward higher burden in higher-SDI settings than as definitive proof of a stable relative pro-rich gradient.

Absolute inequality, assessed by the SII, declined modestly but remained substantial (**Figure 2**, Panel C and D). DALY SII decreased from 143.34 per 100 000 (95% CI = 90.09, 196.58) in 1990 to 136.55 (95% CI = 100.25, 172.85) in 2021, while YLD SII declined from 236.48 (95% CI = 196.51, 276.44) to 214.13 (95% CI = 186.48, 241.77). These findings suggest that although absolute inequality narrowed slightly over time, sizeable cross-country gaps in burden persisted.

### Frontier analysis

Frontier benchmarking showed substantial descriptive efficiency gaps in 2021 (**Figure 2**, Panel E and F). Most countries had higher rates than their frontier values at the same SDI, with the largest excesses observed in several high-SDI settings. For DALYs, Belgium and Andorra showed the largest gaps, whereas low-SDI countries such as Niger and Somalia were close to the frontier. A similar pattern was observed for YLDs, with the largest frontier gap in Andorra and minimal gaps in countries such as the Central African Republic, Chad, and Somalia.

### Burden decomposition

Decomposition analysis showed that global increases in absolute burden from 1990 to 2021 were driven mainly by population growth, with reductions in age-specific rates partly offsetting them (Figure S4, Panels A and B in the **Online Supplementary Document**). For DALYs, population growth explained about 115% of the increase and rate changes about −15%. YLDs showed a similar pattern.

Regional heterogeneity was evident. In many low- and middle-SDI regions, especially South Asia and parts of Latin America, burden growth was driven mainly by population expansion, whereas in some high-SDI settings, including high-income North America and Oceania, rising age-specific rates contributed materially to increases in burden.

### Conditional projections to 2050

BAPC modelling suggested continued evolution of the global burden through 2050 under continuation of recent patterns and United Nations demographic assumptions (Figure S5, Panel A and B in the **Online Supplementary Document**). Age-standardised rates were projected to decline further, with ASDR falling to 212.0 per 100 000 (95% CI = 111.3, 312.6) and ASYR to 171.9 per 100 000 (95% CI = 82.0, 261.7) by 2050. Sensitivity analyses using alternative forecast horizons yielded nearly identical estimates for overlapping years (Table S2 in the **Online Supplementary Document**).

Despite projected declines in age-standardised rates, the absolute burden was expected to increase because of demographic expansion. DALYs were projected to rise from 2.07 million in 2021 to 2.29 million in 2050, and YLDs from 1.40 million to 1.86 million. These estimates should be interpreted as conditional projections rather than fixed predictions, and uncertainty widened substantially with longer forecast horizons.

### Genetic triangulation analyses

**Figure 3:**
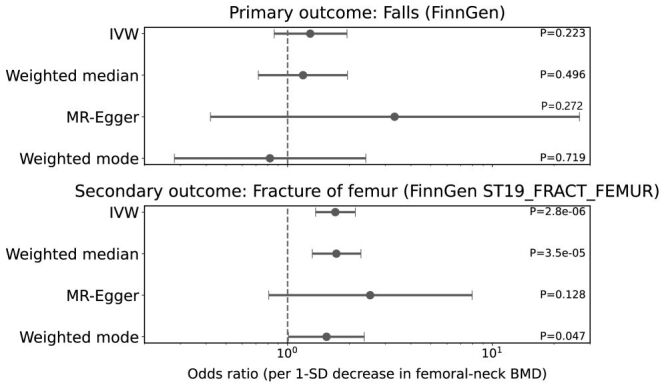
Genetic triangulation using two-sample Mendelian randomisation. Forest plots show Mendelian randomisation estimates of genetically predicted lower femoral-neck bone mineral density (FN-BMD) on medically recorded falls (primary; FinnGen FALLS) and fracture of femur (secondary; FinnGen ST19_FRACT_FEMUR). Points indicate odds ratios (ORs) per 1-SD decrease in FN-BMD and horizontal lines indicate 95% confidence intervals. Estimates are shown for inverse-variance-weighted, weighted-median, MR-Egger, and weighted-mode methods; the dashed line marks the null (OR = 1). CI – confidence interval, FN-BMD – femoral-neck bone mineral density, IVW – inverse-variance weighted, MR – Mendelian randomisation, OR – odds ratio, SD – standard deviation.

Using 21 independent instruments for femoral-neck BMD (mean F-statistic = 51.7; minimum F-statistic = 31.5) (Table S3 in the **Online Supplementary Document**), genetically predicted lower BMD showed no clear association with medically recorded falls (IVW OR per 1-SD decrease = 1.29; 95% CI = 0.86, 1.95, *P* = 0.223) (**Figure 3**, **Table 2**). Sensitivity analyses were directionally similar, heterogeneity was present, and there was no evidence of directional pleiotropy (Table S4 in the **Online Supplementary Document**).

**Table 2:** Two-sample Mendelian randomisation estimates of the effect of femoral-neck BMD on falls and fracture of the femur*

Outcome and method	Method	SNPs, n	OR (95% CI)	*P*-value
Falls (FinnGen)				
*IVW*		21	1.29 (0.86, 1.95)	0.223
*Weighted median*		21	1.19 (0.72, 1.96)	0.496
*MR-Egger*		21	3.33 (0.42, 26.64)	0.272
*Weighted mode*		21	0.82 (0.28, 2.41)	0.719
Fracture of femur (ST19_FRACT_FEMUR)				
*IVW*		21	1.71 (1.37, 2.14)	2.78 × 10^−6^
*Weighted median*		21	1.73 (1.32, 2.28)	3.54 × 10^−5^
*MR-Egger*		21	2.53 (0.81, 7.97)	0.128
*Weighted mode*		21	1.55 (1.01, 2.37)	0.047

CI – confidence interval, IVW – inverse-variance weighted, OR – odds ratio, SNP – single-nucleotide polymorphism.

*All estimates are expressed as OR per 1-SD decrease in genetically predicted femoral-neck BMD. Outcomes were ascertained from FinnGen release R10.

In secondary analyses, genetically predicted lower femoral-neck BMD was associated with higher risk of fracture of femur (IVW OR per 1-SD decrease = 1.71; 95% CI = 1.37, 2.14; *P* = 2.78 × 10^−6^), with broadly consistent weighted-median and weighted-mode estimates (**Figure 3**, **Table 2**). Heterogeneity was modest and the MR-Egger intercept did not indicate directional pleiotropy; additional diagnostics are shown in Figure S6–S9 and Table S4 in the **Online Supplementary Document**. Overall, the MR pattern was more compatible with fracture susceptibility after falls than with a strong direct effect on fall occurrence.

## DISCUSSION

### Principal findings

From 1990 to 2021, the global LBMD-attributable fall-related injury burden in women aged 50–69 years increased markedly in absolute terms, whereas age-standardised rates declined only modestly. Women consistently carried a higher burden than men of the same age. Higher observed burden clustered in several high-SDI settings, and both absolute inequality and frontier gaps remained substantial. Conditional projections suggest that demographic change will sustain growth in the absolute disability burden even if age-standardised rates continue to decline, while complementary MR analyses support a stronger link with fracture susceptibility than with fall occurrence.

### Mechanisms and comparison with previous studies

Compared with prior GBD 2021 reports on midlife falls [15] and the comparative risk assessment of major risk factors [16], our study specifically focuses on women aged 50–69 years and extends the evidence to inequality, frontier benchmarking, decomposition, and conditional forecasting. A central interpretive point is that the GBD-attributed burden should not be read as proving that LBMD directly increases the probability of falling. Rather, the clinical meaning of these estimates is more plausibly that lower bone density amplifies the chance that a fall will result in fracture, prolonged recovery, and disability. This distinction also helps reconcile the apparently discordant findings from the two analytic components. A population can show substantial LBMD-attributable fall-related disability within the GBD framework even if genetically predicted lower BMD does not materially increase fall occurrence, because much of the attributable burden may arise from the greater likelihood that a fall leads to fracture, functional loss, and prolonged recovery.

It may seem counterintuitive that higher-SDI countries show greater observed LBMD-attributable fall-related injury burden. Several explanations are plausible. Women in higher-SDI settings more often survive into later midlife with sufficient longevity to accumulate skeletal fragility and to live long enough with nonfatal disability after fractures [2,8,39]. In addition, differences in imaging access, case ascertainment, trauma care, rehabilitation, and coding practices may make the nonfatal burden more visible in well-resourced settings. These findings, therefore, should not be interpreted as indicating that socioeconomic development per se worsens bone health, but rather that development shapes exposure recognition, survival, and disability expression.

The observed geography is broadly consistent with prior evidence that hip and other fragility fracture rates have historically been highest in Northern Europe and North America and lower in many parts of Asia and Africa [8,40]. A recent cohort study also noted substantial geographic and ethnic disparities in osteoporotic fracture incidence, with populations of European ancestry at elevated risk [41]. Our analysis refines these patterns by focusing on women aged 50–69 years and by emphasising nonfatal disability in addition to total health loss.

The modest declines in age-standardised rates may suggest gradual improvement in osteoporosis management, fracture prevention, and post-injury care, but the magnitude of improvement remains limited [42]. In some higher-income regions, early gains from osteoporosis treatment may have been offset by later stagnation in screening and treatment persistence, while obesity, diabetes, dual-energy x-ray absorptiometry (DXA) availability, fracture liaison services, rehabilitation access, and coding intensity may also contribute to cross-country differences [43–46]. Because these estimates are model-based and the outcome is multifactorial, such temporal patterns should be interpreted cautiously rather than as direct evidence that falling itself has become less biologically linked to LBMD.

This age-specific focus is important because substantial disability is already evident before age 70, particularly in the late 50s and 60s, when preventive intervention may still avert later-life fracture cascades [42]. These findings are also consistent with the concept of osteosarcopenia and muscle-bone crosstalk. In women aged 50–69 years, clinically meaningful musculoskeletal ageing appears to be under way during the menopausal transition and early post menopause, and disability after falls is shaped not only by skeletal fragility but also by muscle strength, balance, and physical performance [5,7,11,16]. This supports integrated prevention that combines osteoporosis identification and treatment with exercise-based strategies to preserve function and reduce fracture-related disability.

The MR analyses help clarify the apparent tension between GBD attribution and individual-level causal inference. In FinnGen, genetically predicted lower femoral-neck BMD was not clearly associated with medically recorded falls, yet it was robustly associated with fracture of the femur. This pattern fits the interpretation that LBMD is more strongly related to fracture susceptibility and disability after falls than to fall occurrence itself. At the same time, MR and GBD are not directly comparable: MR reflects lifelong genetic liability in predominantly European-ancestry populations with registry phenotypes, whereas GBD estimates age- and sex-specific population burden under a comparative risk assessment framework [16,47,48]. The value of MR here is therefore interpretive rather than confirmatory. It helps narrow the most biologically coherent reading of the GBD estimates without implying that the two approaches should yield numerically parallel effects.

### Clinical and policy implications

In high-SDI settings, where observed burden and frontier gaps are large, health systems should strengthen earlier osteoporosis risk assessment, timely access to DXA, secondary fracture prevention, and rehabilitation planning. Current guidelines support screening women aged 65 years or older and younger women at elevated risk [2], but our findings suggest that meaningful disability is already accumulating in the late 50s and early 60s. Earlier risk assessment in high-risk women may therefore be justified before age 65. Fall-prevention strategies should be integrated with bone-health strategies rather than treated separately, with the practical goal of both reducing fall exposure and reducing the probability that a fall results in fracture-related disability [13,49–51].

In lower-SDI settings, currently lower attributed rates should not obscure the likelihood of substantial future growth in absolute fracture-related disability as populations age and expand. Investment in bone health, case finding, nutrition, mobility promotion, community-based prevention, and post-fracture rehabilitation should begin before these burdens escalate. Health systems will also need to prepare for continuing growth in non-fatal disability, including rehabilitation services, assistive technologies, and coordinated fracture-aftercare pathways.

### Limitations

This study also has several limitations. First, GBD estimates are model-based and may be affected by cross-country heterogeneity in case ascertainment, osteoporosis screening, and fracture surveillance. Second, the National Health and Nutrition Examination Survey (NHANES)-derived reference is a standardised GBD comparator rather than a universally representative normative BMD distribution, so alternative reference distributions would mainly affect the absolute magnitude of attributable estimates. Third, PAF-based attribution in a multifactorial outcome such as falls should not be interpreted as direct individual-level causality and likely reflects fracture susceptibility and post-fall disability more strongly than fall occurrence itself. Fourth, the projections are conditional rather than deterministic, and the frontier analysis is descriptive rather than causal. Finally, the MR analyses were based largely on European-ancestry registry data and were not restricted to women aged 50–69 years, limiting direct comparability with the GBD estimates.

## CONCLUSIONS

In women aged 50–69 years, LBMD-attributable fall-related injury burden within the GBD framework remains substantial and unequally distributed despite modest declines in age-standardised rates since 1990. The combined GBD and MR evidence suggests that the main role of LBMD is more likely to lie in fracture susceptibility and disability after falls than in fall occurrence itself. Large frontier gaps indicate opportunities for improvement in osteoporosis detection, treatment, rehabilitation, and integrated fall-prevention strategies. Even if age-specific risks continue to decline, population ageing and growth are likely to increase the absolute number of women living with fracture-related disability by 2050.

## Additional material


Online Supplementary Document
