# Supplementary material for: Global burden of fall-related injuries attributable to low bone mineral density in women aged 50–69 years: inequalities, projections to 2050, and Mendelian randomisation
Source: J Glob Health. 2026 Jun 19;16:04180. doi: 10.7189/jogh.16.04180 (PMC13280865; doi:10.7189/jogh.16.04180)
Supplement: Online Supplementary Document [file jogh-16-04180-s001.pdf]

Supplement to: Zhang B, Chen Q, Chen Y, Zhang H. Global burden of fall-related injuries attributable to low bone mineral density in women aged 50–69 years: inequalities, projections to 2050, and Mendelian randomisation. J Glob Health. 2026;16:04180.

Content

- **Table S1.** DALYs and YLDs due to LBMD-attributable fall-related injury burden among women aged 50-69 years, 1990 and 2021.
- **Table S2.** Sensitivity of BAPC projections to alternative forecast horizons (2035, 2040, and 2050).
- **Table S3.** Summary of genetic instruments for femoral-neck BMD.
- **Table S4.** Sensitivity analyses and pleiotropy diagnostics for Mendelian randomization.
- **Table S5.** Adherence to JoGH's Guidelines for Reporting Analyses of Big Data Repositories Open to the Public (GRABDROP).
- **Table S6.** STROBE checklist.
- **Figure S1.** Global and temporal patterns of fall-related burden attributable to major risks among women aged 50–69 years, 1990–2021 (GBD 2021).
- **Figure S2.** Global sex- and age-specific fall-related injury burden attributable to low bone mineral density, 1990–2021.
- **Figure S3.** Socio-demographic gradient (SDI) in LBMD-attributable fall-related injury burden in women aged 50–69 years.
- **Figure S4.** Decomposition of changes in low bone mineral density (LBMD)-attributable fall-related injury burden among women aged 50–69 years, 1990–2021.
- **Figure S5.** Conditional projections of LBMD-attributable fall-related injury burden in women aged 50–69 years, 1990–2050.

- **Figure S6.** Single-SNP and pooled MR estimates of femoral-neck BMD.
- **Figure S7.** Funnel plots for MR analyses of femoral-neck BMD.
- **Figure S8.** Leave-one-out sensitivity analyses for the MR effect of femoral-neck BMD.
- **Figure S9.** Scatter plots of SNP-exposure versus SNP-outcome associations for femoral-neck BMD.

Table S1. DALYs and YLDs due to LBMD-attributable fall-related injury burden among women aged 50-69 years, 1990 and 2021.

Location	DALYs 1990 (95% UI)	DALYs 2021 (95% UI)	YLDs 1990 (95% UI)	YLDs 2021 (95% UI)
Global	1,083,598 (853,310 to 1,343,612)	2,091,475 (1,639,042 to 2,629,110)	737,417 (519,562 to 989,220)	1,414,595 (989,690 to 1,907,214)
High SDI	285,667 (207,223 to 377,402)	484,332 (351,613 to 646,515)	244,558 (169,054 to 336,637)	418,153 (287,771 to 580,601)
High-middle SDI	266,821 (200,524 to 343,791)	401,404 (298,496 to 533,933)	218,631 (153,803 to 296,200)	332,996 (228,969 to 460,505)
Middle SDI	242,257 (195,029 to 291,808)	596,696 (463,697 to 742,501)	145,531 (104,619 to 191,657)	381,366 (270,728 to 513,083)
Low-middle SDI	221,853 (174,186 to 268,750)	468,393 (375,790 to 561,276)	103,131 (73,722 to 135,412)	222,323 (160,840 to 293,711)
Low SDI	65,744 (52,570 to 79,308)	139,246 (112,751 to 168,205)	24,620 (17,742 to 32,437)	58,658 (42,416 to 77,073)
Andean Latin America	2,332 (1,829 to 2,853)	7,066 (5,457 to 8,898)	1,530 (1,100 to 2,025)	4,934 (3,460 to 6,695)
Australasia	5,900 (4,110 to 8,086)	12,915 (8,976 to 17,630)	5,420 (3,634 to 7,624)	11,801 (7,908 to 16,453)
Caribbean	2,667 (2,104 to 3,270)	6,195 (4,816 to 7,770)	1,700 (1,216 to 2,300)	4,235 (2,999 to 5,714)
Central Asia	8,610 (6,443 to 11,045)	11,997 (8,829 to 15,829)	6,920 (4,824 to 9,337)	10,078 (6,966 to 13,804)
Central Europe	54,720 (41,914 to 69,652)	45,364 (32,935 to 60,564)	42,833 (30,325 to 57,566)	38,757 (26,634 to 54,018)
Central Latin America	20,265 (15,789 to 25,465)	38,273 (28,727 to 48,970)	15,529 (11,171 to 20,705)	31,435 (21,960 to 42,094)
Central Sub-Saharan Africa	4,739 (3,667 to 5,979)	10,943 (8,415 to 14,128)	1,430 (1,038 to 1,877)	3,713 (2,704 to 4,910)
East Asia	172,026 (132,457 to 219,184)	402,019 (301,251 to 528,545)	123,835 (88,870 to 165,274)	317,285 (219,881 to 438,513)
Eastern Europe	94,479 (69,331 to 124,728)	92,788 (67,923 to 124,515)	82,026 (57,131 to 112,024)	78,395 (53,680 to 110,262)
Eastern Sub-Saharan Africa	15,243 (11,789 to 19,113)	28,904 (23,515 to 34,861)	4,467 (3,261 to 5,910)	10,022 (7,271 to 13,264)
High-income Asia Pacific	46,506 (32,953 to 62,669)	57,526 (40,087 to 78,792)	41,747 (28,445 to 57,855)	53,394 (36,345 to 74,633)
High-income North America	71,280 (51,106 to 95,165)	194,245 (142,954 to 263,042)	62,840 (43,343 to 86,969)	162,955 (112,525 to 231,332)
North Africa and Middle East	22,370 (16,904 to 28,763)	55,617 (41,115 to 72,655)	16,177 (11,431 to 21,748)	44,840 (31,059 to 62,149)

Oceania	581 (438 to 756)	1,803 (1,354 to 2,375)	443 (319 to 592)	1,479 (1,072 to 1,981)
South Asia	283,338 (220,002 to 342,926)	667,912 (528,945 to 795,896)	122,266 (87,615 to 160,552)	300,230 (218,365 to 397,178)
Southeast Asia	59,831 (46,427 to 72,149)	124,884 (99,636 to 152,241)	28,050 (20,158 to 37,191)	67,425 (48,574 to 90,043)
Southern Latin America	8,778 (6,490 to 11,313)	15,271 (10,877 to 20,435)	7,534 (5,300 to 10,049)	13,853 (9,464 to 18,989)
Southern Sub-Saharan Africa	1,968 (1,520 to 2,518)	3,548 (2,720 to 4,556)	1,391 (995 to 1,882)	2,330 (1,655 to 3,188)
Tropical Latin America	22,015 (16,526 to 27,953)	56,105 (43,098 to 70,715)	18,264 (12,947 to 24,138)	43,141 (30,568 to 57,676)
Western Europe	172,317 (124,680 to 229,462)	225,522 (160,083 to 302,975)	147,786 (101,629 to 204,342)	200,432 (136,288 to 277,465)
Western Sub-Saharan Africa	13,634 (10,944 to 16,160)	32,578 (26,105 to 39,818)	5,230 (3,810 to 6,879)	13,858 (10,062 to 18,271)

Note: DALYs=Disability-Adjusted Life Years; LBMD=Low bone mineral density; GBD=Global Burden of Disease; SDI=socio-demographic index; UI=uncertainty interval; YLDs=Years Lived with Disability

Table S2. Sensitivity of BAPC projections to alternative forecast horizons (2035, 2040, and 2050).

Panel A. Overlapping-year posterior means across forecast horizons

Metric	Year	2035 horizon	2040 horizon	2050 horizon
ASDR	2035	246.9080	246.9081	246.9125
ASDR	2040	—	234.6540	234.6620
ASYR	2035	177.1600	177.1580	177.1610
ASYR	2040	—	175.3705	175.3764

Panel B. Terminal-year posterior means and 95% credible intervals

Metric	Horizon	Terminal-year estimate	95% CrI	Width
ASDR	2035	246.9080	218.3790–275.4369	57.0578
ASDR	2040	234.6540	186.5140–282.7940	96.2799
ASDR	2050	211.9575	111.2736–312.6413	201.3676
ASYR	2035	177.1600	153.6942–200.6258	46.9315
ASYR	2040	175.3705	134.8813–215.8598	80.9785
ASYR	2050	171.8631	82.0260–261.7002	179.6742

Note: Across overlapping years, posterior means were nearly identical across the 2035, 2040, and 2050 forecast-horizon models. Uncertainty widened materially as the forecast horizon lengthened, supporting cautious interpretation of the 2050 estimates as conditional projections rather than deterministic forecasts.

Table S3. Summary of genetic instruments for femoral-neck BMD.

Metric	Value
No. of instruments	21
Mean F statistic	51.7

Metric	Value
Median F statistic	46.0
Minimum F statistic	31.5

Instruments were selected from a genome-wide association study of femoral-neck bone mineral density (BMD). All 21 SNPs were independent ($r^2 < 0.001$, 10-Mb clumping window) and genome-wide significant ($P < 5\times10^{-8}$). F statistics were estimated as $(beta/s.e.)^2$ and are a measure of instrument strength; $F > 10$ indicates an adequate instrument. SNP, single-nucleotide polymorphism.

Table S4. Sensitivity analyses and pleiotropy diagnostics for Mendelian randomization.

Outcome	Test	Statistic	df	s.e.	P value
Falls (FinnGen)	Cochran's Q (IVW)	31.99	20	—	0.043
	Cochran's Q (MR-Egger)	30.65	19	—	0.044
	MR-Egger intercept	0.060	—	0.066	0.375
Fracture of femur (ST19_FRACT_FEMUR)	Cochran's Q (IVW)	29.94	20	—	0.071
	Cochran's Q (MR-Egger)	29.21	19	—	0.063
	MR-Egger intercept	0.025	—	0.036	0.501

Cochran's Q statistics assess between-instrument heterogeneity. The MR-Egger intercept tests for directional (unbalanced) horizontal pleiotropy; a non-significant intercept indicates no evidence of systematic bias. s.e., standard error; df, degrees of freedom; —, not applicable.

Table S5. Adherence to JoGH's Guidelines for Reporting Analyses of Big Data Repositories Open to the Public (GRABDROP).

JoGH GRABDROP item	Authors' response
1. Please list all papers published by each co-author in previous three years that were based on secondary analysis of a big data repository.	Within the previous three years, the authors have one related secondary analysis of a big data repository: Zhang B, Chen Q, Chen Y, Zhang H. Global burden and inequalities of rheumatoid arthritis in adults aged 15–49 years from 1990 to 2021 and projections to 2050: a cross-sectional analysis from Global Burden of Disease Study

JoGH GRABDROP item	Authors' response
	2021. Clinical Rheumatology. Published online 7 May 2026. doi:10.1007/s10067-026-08158-z. That study addressed rheumatoid arthritis in adults aged 15–49 years, whereas the present study focuses on low bone mineral density-attributable fall-related injury burden in women aged 50–69 years and adds inequality, frontier, decomposition, projection, and Mendelian randomisation triangulation analyses.
2. Please explain the key elements of your study design and the use of the available datasets that make your study an original scientific contribution.	This study combines GBD 2021 comparative risk assessment estimates for low bone mineral density-attributable fall-related injury burden in women aged 50–69 years with temporal trend analysis, socioeconomic inequality metrics, frontier benchmarking, Das Gupta decomposition, Bayesian age-period-cohort projections to 2050, and two-sample Mendelian randomisation using femoral-neck bone mineral density instruments and FinnGen outcomes. The key originality lies in distinguishing fall occurrence from fracture susceptibility/post-fall disability and in focusing on a clinically actionable midlife female population.
3. Please list all publications that addressed similar research questions in the same dataset and indicate where you cited them in your paper.	The manuscript cites relevant GBD and falls/fracture publications, including the GBD 2019 fracture study (reference 8), the GBD ageing study (reference 12), the GBD 2021 risk factors study (reference 16), the GBD 2021 diseases and injuries study (reference 19), the GBD 2021 low bone mineral density study (reference 20), and a GBD 2021 analysis of falls among midlife women (reference 15). These are discussed in the Introduction and Discussion to position the present analysis relative to prior work.
4. Please explain how you addressed multiple testing through an appropriately rigorous statistical threshold and indicate this in the methods section.	The epidemiological analyses were prespecified, descriptive, and interpreted using uncertainty intervals rather than as a large exploratory hypothesis-screening exercise. For Mendelian randomisation, the falls outcome was prespecified as primary and fracture of femur as secondary; inverse-variance weighted analysis was primary, with MR-Egger, weighted median, weighted mode, heterogeneity, pleiotropy, leave-one-out, and instrument-strength diagnostics as sensitivity analyses. Two-sided $P < 0.05$ was specified, and results were interpreted with emphasis on effect estimates, confidence intervals, and biological coherence rather than isolated nominal significance.
5. Please declare to what extent AI chatbots have been used in developing your paper and to which parts of the paper they contributed.	AI assistance was used only for language polishing. AI was not used for data acquisition, statistical analyses, figure generation, interpretation of results, or scientific conclusions. All authors reviewed and approved the final submitted text and remain fully responsible for the manuscript content.

Table S6. STROBE checklist.

STROBE item	Recommendation	Manuscript location / response
1(a)	Indicate the study's design with a commonly used term in the title or	Abstract and Methods: GBD 2021 estimates and two-sample Mendelian

STROBE item	Recommendation	Manuscript location / response
	abstract.	randomisation are described; the retrospective observational study design is stated in the Methods.
1(b)	Provide an informative and balanced summary of what was done and what was found.	Abstract: Background, Methods, Results, and Conclusions.
2	Explain the scientific background and rationale for the investigation.	Introduction, paragraphs 1–4.
3	State specific objectives, including any prespecified hypotheses.	Introduction, final paragraph.
4	Present key elements of study design early in the paper.	Methods: Study design and data source.
5	Describe setting, locations, and relevant dates, including recruitment, exposure, follow-up, and data collection periods.	Methods: Study design and data source; GBD 1990–2021 estimates; projections to 2050; FinnGen Release 10 outcomes.
6(a)	Give eligibility criteria and the sources and methods of selection of participants.	Methods: Study design and data source; women aged 50–69 years from GBD 2021, with men used for contextual comparison; MR datasets described separately.
6(b)	For matched studies, give matching criteria and number of exposed/unexposed.	Not applicable; this was not a matched individual-level study.
7	Clearly define all outcomes, exposures, predictors, potential confounders, and effect modifiers.	Methods: Exposure and outcome definitions; outcome measures; socioeconomic inequality metrics; MR section.
8	For each variable of interest, give sources of data and details of methods of assessment.	Methods: GBD 2021 source, LBMD definition, ICD-10 falls definition, FinnGen outcomes, and OpenGWAS instruments.
9	Describe any efforts to address potential sources of bias.	Methods and Limitations: GBD uncertainty, MR sensitivity analyses, heterogeneity/pleiotropy diagnostics, and interpretation limits.
10	Explain how the study size was arrived at.	Methods: all available GBD 2021 estimates for 204 countries/territories and eligible age-sex strata; MR used 21 independent instruments.
11	Explain how quantitative variables were handled in the analyses.	Methods: age-standardised rates, counts, SDI rankings, EAPC, concentration index, slope index, frontier gaps, decomposition, and BAPC projections.
12(a)	Describe all statistical methods, including those used to control for confounding.	Methods: Statistical analysis subsections; confounding control was not individual-level because GBD estimates are aggregated/modelled.
12(b)	Describe methods used to examine subgroups and interactions.	Methods and Results: global, regional, national, SDI quintile, sex-contextual, and age-specific analyses.
12(c)	Explain how missing data were addressed.	Methods: analyses used publicly available modelled GBD/FinnGen

STROBE item	Recommendation	Manuscript location / response
		summary data; uncertainty intervals and MR diagnostics reported.
12(d)	If applicable, describe analytical methods taking account of sampling strategy.	Not applicable to primary analyses; GBD estimates are produced using established GBD modelling and uncertainty draws.
12(e)	Describe sensitivity analyses.	Methods: restricted cubic splines/model fit, projection horizon sensitivity, MR-Egger/weighted median/weighted mode, heterogeneity, pleiotropy, leave-one-out, and instrument strength.
13(a)	Report numbers of individuals at each stage of study.	Not applicable to aggregated GBD analysis; Results report global/national burden estimates; MR reports 21 genetic instruments.
13(b)	Give reasons for non-participation at each stage.	Not applicable; no direct participant recruitment.
13(c)	Consider use of a flow diagram.	Not applicable; no individual-level participant flow.
14(a)	Give characteristics of study participants and exposures.	Results: sex- and age-specific patterns; geographic variations; SDI gradients.
14(b)	Indicate number of participants with missing data for each variable.	Not applicable; aggregated/modelled estimates were used.
15	Report numbers of outcome events or summary measures.	Results: global temporal trends, absolute DALYs/YLDs, rates, MR estimates, Tables 1–2 and Supplementary Tables.
16(a)	Give unadjusted and, if applicable, confounder-adjusted estimates and precision.	Results: age-standardised rates with uncertainty intervals; MR odds ratios with confidence intervals.
16(b)	Report category boundaries when continuous variables were categorized.	Methods/Results: SDI quintiles and age groups; GBD age-standardised rates.
16(c)	Translate estimates of relative risk into absolute risk for a meaningful time period.	Results/Discussion: absolute DALYs/YLDs and projected counts to 2050 complement rate and odds ratio estimates.
17	Report other analyses done.	Results: inequality, frontier analysis, decomposition, projections, and MR sensitivity analyses.
18	Summarise key results with reference to study objectives.	Discussion: Principal findings.
19	Discuss limitations, considering sources of potential bias or imprecision.	Discussion: Limitations.
20	Give a cautious overall interpretation of results.	Discussion and Conclusions.
21	Discuss generalisability of results.	Discussion: Clinical and policy implications; Limitations.
22	Give the source of funding and role of funders.	Funding statement.

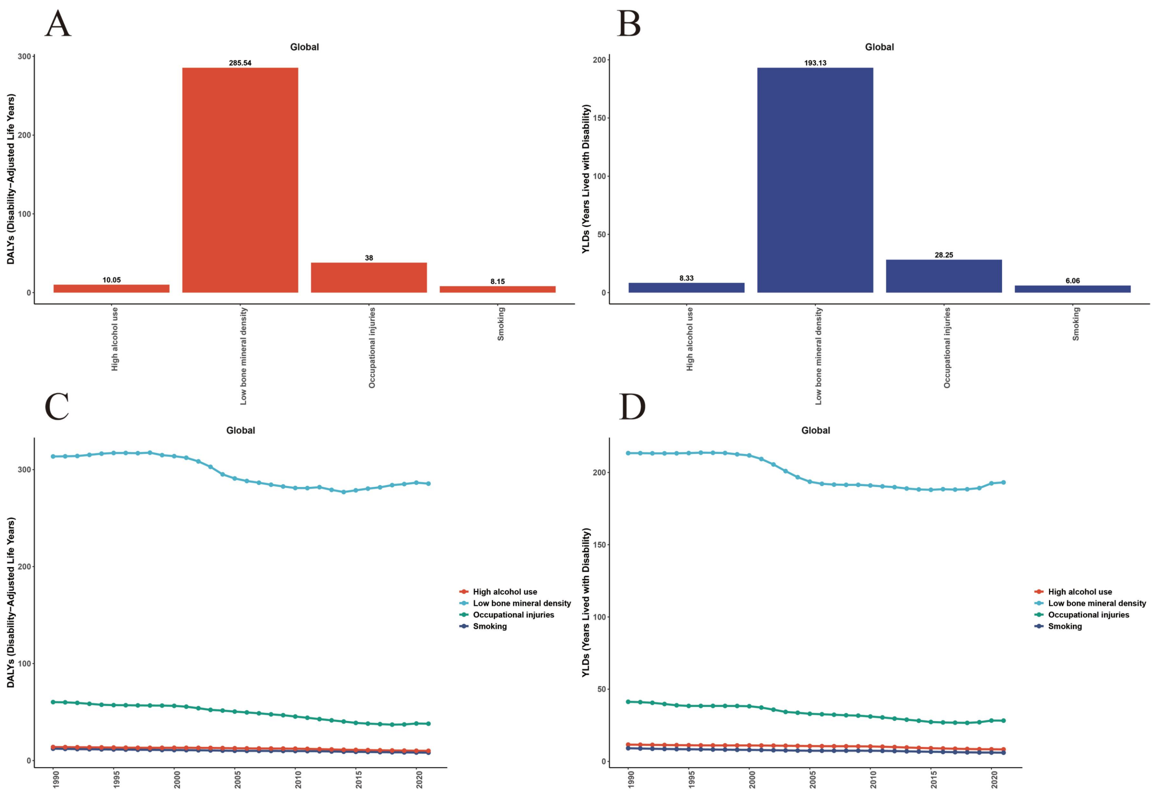

Figure S1. Global and temporal patterns of fall-related burden attributable to major risks among women aged 50–69 years, 1990–2021 (GBD 2021).

Panel A. Age-standardized DALY rates in 2021 attributable to high alcohol use, low bone mineral density (LBMD), occupational injuries, and smoking. Panel B. Age-standardized YLD rates in 2021 for the same risks. **Panel C.** Trends in age-standardized DALY rates, 1990–2021. **Panel D.** Trends in age-standardized YLD rates, 1990–2021. DALY – disability-adjusted life year, LBMD – low bone mineral density, YLD – years lived with disability.

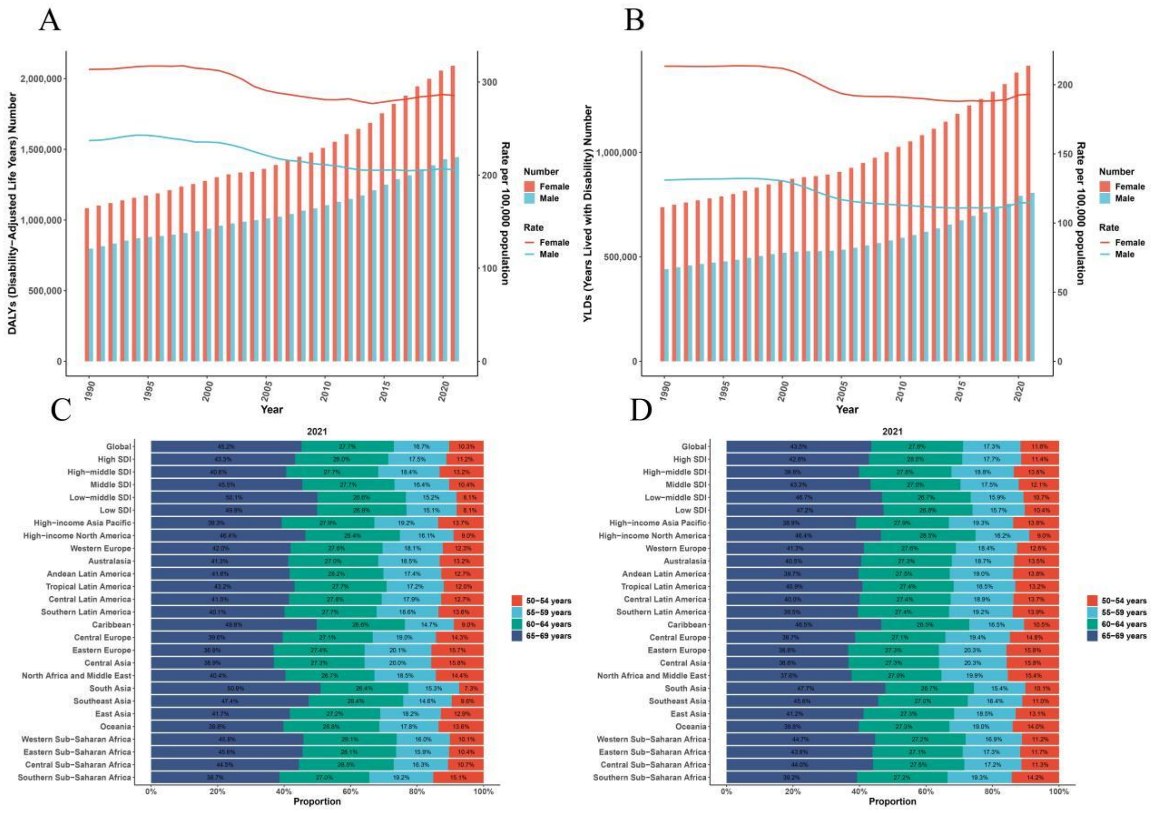

Figure S2. Global sex- and age-specific fall-related injury burden attributable to low bone mineral density, 1990–2021.

Panel A. Global trends in disability-adjusted life years (DALYs) due to LBMD-attributable fall-related injury burden by sex, shown as absolute numbers (bars) and age-standardized rates per 100,000 population (lines). **Panel B.** Global trends in years lived with disability (YLDs) by sex, shown as absolute numbers and age-standardized rates. **Panel C.** Age distribution of DALYs in 2021 across global, SDI, and regional groups. **Panel D.** Age distribution of YLDs in 2021 across SDI and regional groups. DALY – disability-adjusted life year, LBMD – low bone mineral density, SDI – socio-demographic index, YLD – years lived with disability.

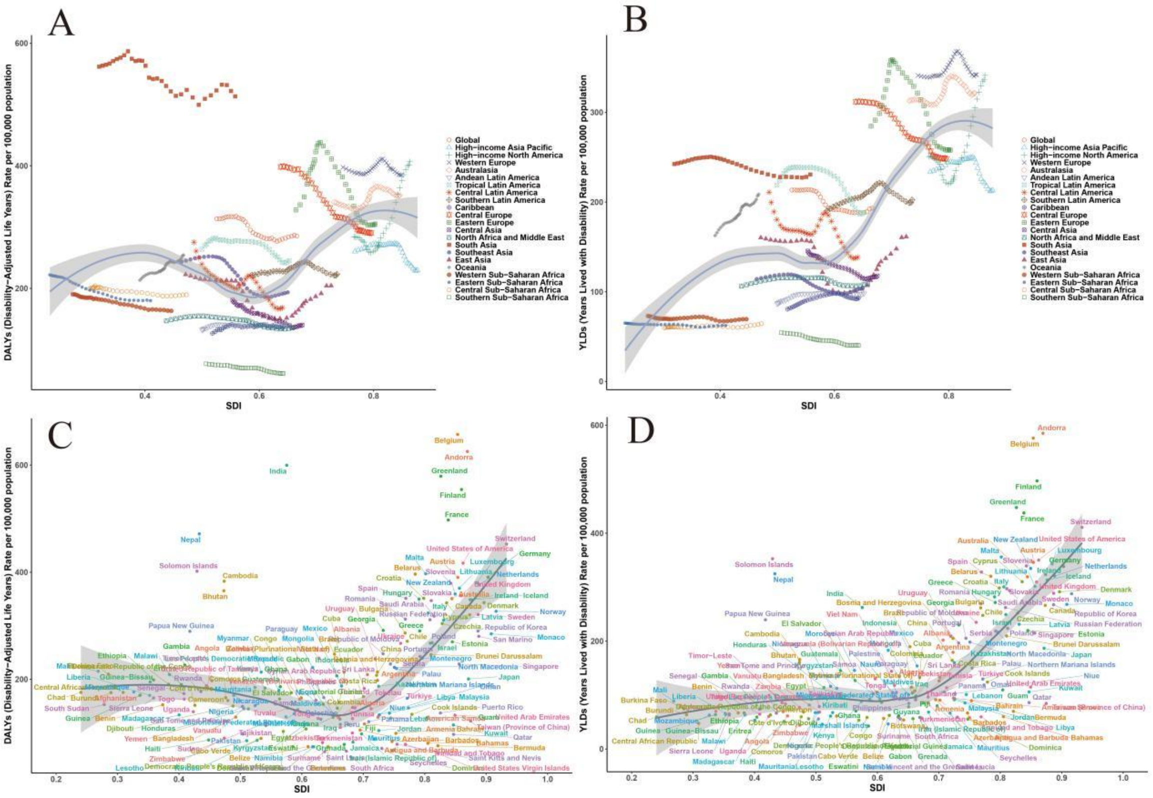

Figure S3. Socio-demographic gradient (SDI) in LBMD-attributable fall-related injury burden in women aged 50–69 years.

Panel A. Regional trajectories of age-standardized DALY rates (ASDR) versus SDI, 1990–2021. **Panel B.** Regional trajectories of age-standardized YLD rates (ASYR) versus SDI, 1990–2021. **Panel C.** Country-level ASDR versus SDI in 2021. **Panel D.** Country-level ASYR versus SDI in 2021. ASDR – age-standardized disability-adjusted life year rate, ASYR – age-standardized years lived with disability rate, DALY – disability-adjusted life year, SDI – socio-demographic index, YLD – years lived with disability.

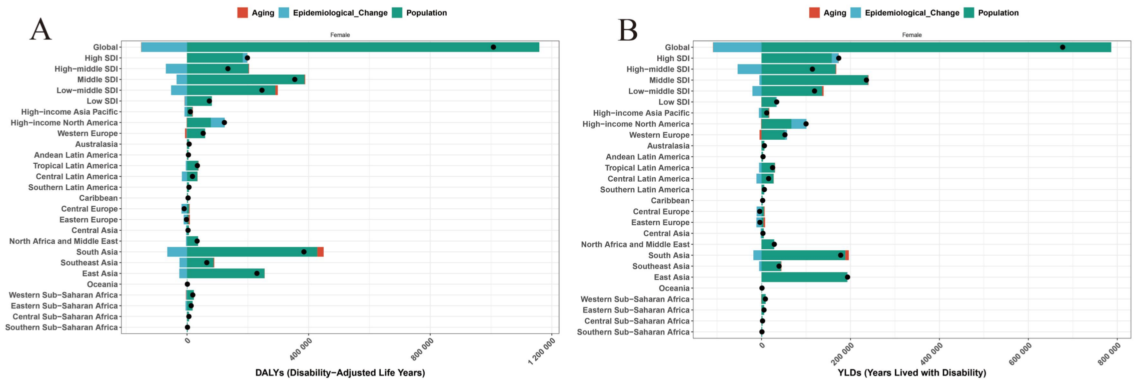

Figure S4. Decomposition of changes in low bone mineral density (LBMD)-attributable fall-related injury burden among women aged 50–69 years, 1990–2021. Panel A. Global and regional changes in DALYs attributable to population growth, ageing, and epidemiological shifts. **Panel B.** Corresponding changes in years lived with disability (YLDs). DALY – disability-adjusted life year, LBMD – low bone mineral density, YLD – years lived with disability.

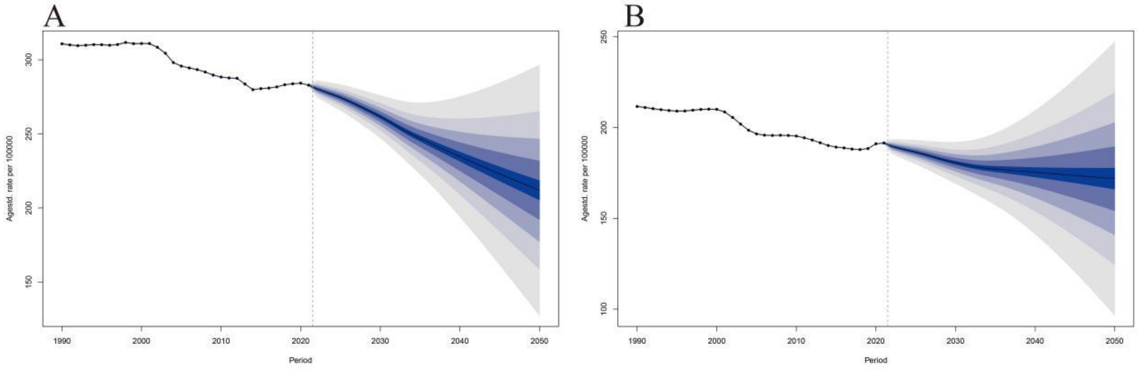

Figure S5. Conditional projections of LBMD-attributable fall-related injury burden in women aged 50–69 years, 1990–2050.

Panel A. Age-standardized DALY rate per 100,000 population. **Panel B.** Age-standardized YLD rate per 100,000 population. Solid lines show Bayesian age-period-cohort posterior means, and shaded ribbons indicate 50%, 80%, and 95% credible intervals. The vertical dashed line marks the transition from fitted values (1990–2021) to projections (2022–2050). BAPC – Bayesian age-period-cohort, CrI – credible interval, DALY – disability-adjusted life year, LBMD – low bone mineral density, YLD – years lived with disability.

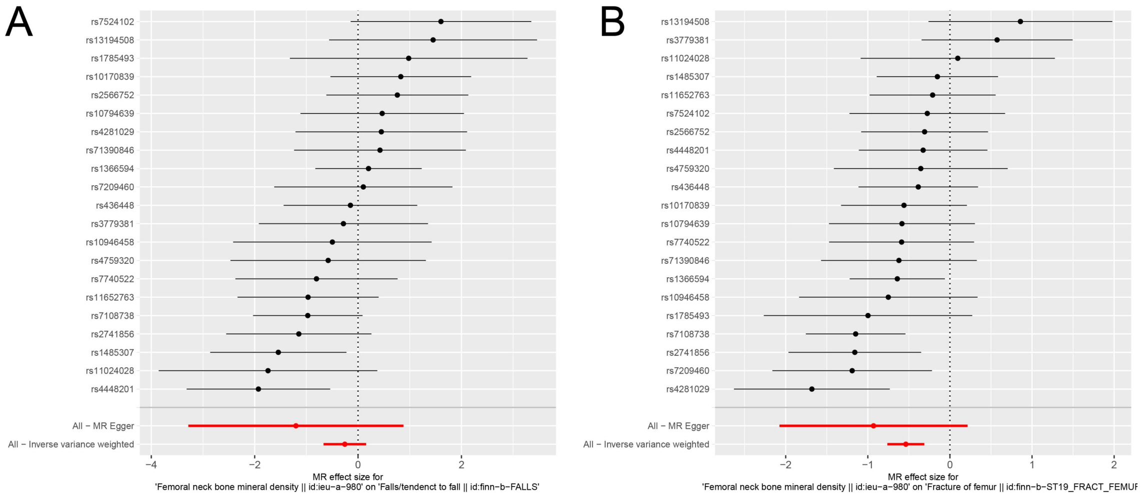

Figure S6. Single-SNP and pooled MR estimates of femoral-neck BMD.

Panel A. Falls. **Panel B.** Fracture of femur. Forest plots show Wald ratio estimates for each genetic instrument (rsID) with 95% confidence intervals (black points and horizontal lines). Red points and lines indicate pooled estimates from the inverse-variance weighted and MR-Egger methods. The dashed line denotes the null effect (β = 0 on the log-odds scale). The exposure was femoral-neck bone mineral density (FN-BMD; GWAS id ieu-a-980), and the outcomes were FinnGen medically recorded falls (FALLS) and fracture of femur (ST19_FRACT_FEMUR). FN-BMD – femoral-neck bone mineral density, IVW – inverse-variance weighted, MR – Mendelian randomization.

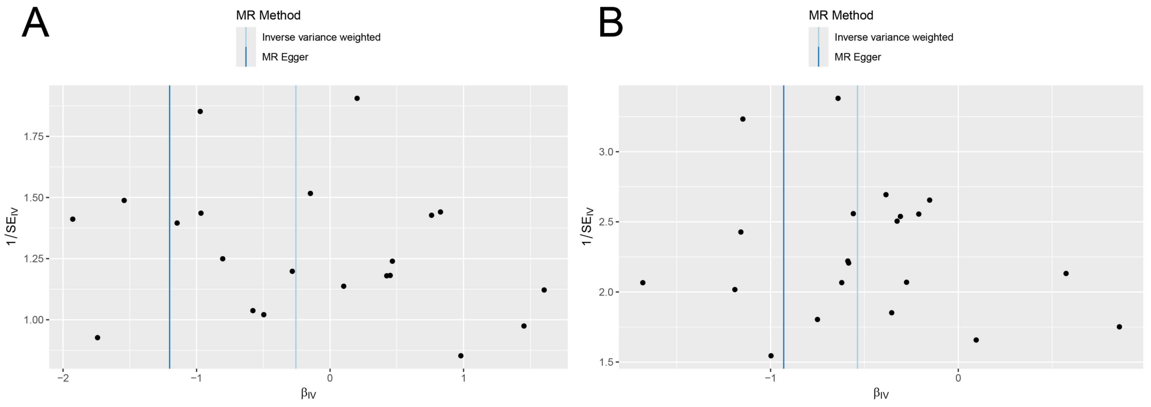

Figure S7. Funnel plots for MR analyses of femoral-neck BMD.

Panel A. Falls. **Panel B.** Fracture of femur. Each point represents a single-SNP Wald ratio estimate (βIV) plotted against its precision (1/SEIV). Vertical lines indicate the pooled estimates from inverse-variance weighted and MR-Egger methods. Visual asymmetry may suggest directional horizontal pleiotropy. IVW – inverse-variance weighted, SNP – single-nucleotide polymorphism.

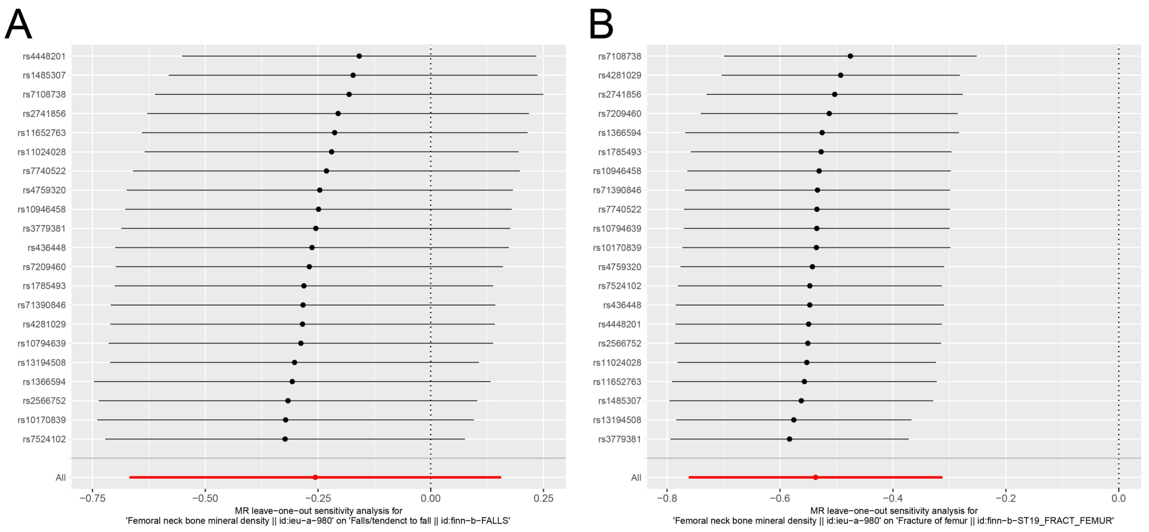

Figure S8. Leave-one-out sensitivity analyses for the MR effect of femoral-neck BMD.

Panel A. Falls. **Panel B.** Fracture of femur. Each point and horizontal line represent the inverse-variance weighted estimate and 95% confidence interval after exclusion of the indicated SNP. The red point and line show the inverse-variance weighted estimate using all instruments. The vertical dashed line indicates the null effect (β = 0). IVW – inverse-variance weighted, SNP – single-nucleotide polymorphism.

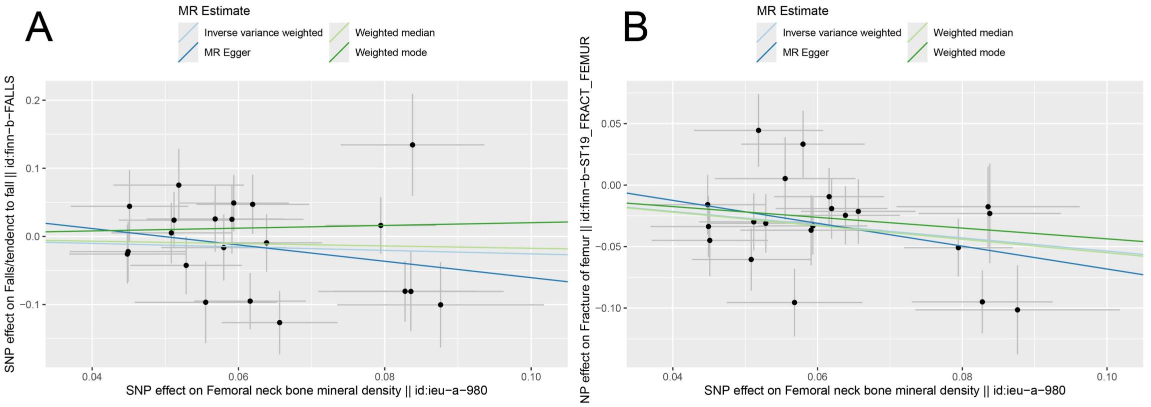

Figure S9. Scatter plots of SNP-exposure versus SNP-outcome associations for femoral-neck BMD.

Panel A. Falls. **Panel B.** Fracture of femur. Points represent SNP-specific associations with standard error bars. Lines correspond to MR estimates derived from inverse-variance weighted, MR-Egger, weighted median, and weighted mode methods. MR – Mendelian randomization, SNP – single-nucleotide polymorphism.
